# Genomic analysis of immunogenic cell death-related subtypes for predicting prognosis and immunotherapy outcomes in glioblastoma multiforme

**DOI:** 10.1515/med-2023-0716

**Published:** 2023-06-02

**Authors:** Zhiye Liu, Wei Li, Guoliang You, Zhihong Hu, Yuji Liu, Niandong Zheng

**Affiliations:** Department of Neurosurgery, The Affiliated Hospital of Southwest Medical University, Luzhou 646000, Sichuan, China; Department of Cerebrovascular Diseases, The People’s Hospital of Leshan City, Leshan 614000, Sichuan, China; Department of Cerebrovascular Diseases, Leshan Shizhong District People’s Hospital, Leshan 614000, Sichuan, China

**Keywords:** glioblastoma multiforme, immunogenic cell death, prognosis, immunotherapy, tumour mutation burden

## Abstract

Immunogenic cell death (ICD), a unique form of cancer cell death, has therapeutic potential in anti-tumour immunotherapy. The aim of this study is to explore the predictive potential of ICD in the prognosis and immunotherapy outcomes of glioblastoma multiforme (GBM). RNA sequencing data and clinical information were downloaded from three databases. Unsupervised consistency clustering analysis was used to identify ICD-related clusters and gene clusters. Additionally, the ICD scores were determined using principal component analysis and the Boruta algorithm via dimensionality reduction techniques. Subsequently, three ICD-related clusters and three gene clusters with different prognoses were identified, with differences in specific tumour immune infiltration-related lymphocytes in these clusters. Moreover, the ICD score was well differentiated among patients with GBM, and the ICD score was considered an independent prognostic factor for patients with GBM. Furthermore, two datasets were used for the external validation of ICD scores as predictors of prognosis and immunotherapy outcomes. The validation analysis suggested that patients with high ICD scores had a worse prognosis. Additionally, a higher proportion of patients with high ICD scores were non-responsive to immunotherapy. Thus, the ICD score has the potential as a biomarker to predict the prognosis and immunotherapy outcomes of patients with GBM.

## 1 Introduction

Glioblastoma multiforme (GBM), a WHO grade IV glioma, is one of the most common primary malignant brain tumours of the central nervous system [1]. Owing to its highly invasive ability, clinical treatment becomes difficult, leading to a high mortality rate. Currently, surgical resection to the maximum extent, followed by radiotherapy and chemotherapy are the standard treatments for GBM [2]. An analysis of large public data from the United States, including more than 40,000 patients with GBM, suggests that the median overall survival (OS) for patients with GBM is only 15 months [3]. Furthermore, data from China suggest that the median OS of patients with GBM is less than 2 years and the 5 year survival rate is less than 10%, indicating a poor prognosis [4,5].

A recent study reported that the central nervous system has an appropriate immune response and is connected with the peripheral immune system [6]. There are functional lymphatic vessels besides the dural vein sinus and the lymphatic vessels that absorb the cerebrospinal fluid and interstitial fluid from the adjacent subarachnoid space. Nonetheless, immune cells can also be present in the lymphatic vessels and communicate with the deep cervical lymph nodes through the skull base foramen [7,8]. The tumour microenvironment is an important factor in the occurrence and development of tumours, for example, tumour-associated macrophages usually promote angiogenesis, inhibit the antitumour function of immune cells, and promote tumour immune escape response [9]. Colony-stimulating factor 1 produced by glioma has been proved to polarise macrophages into the M2 phenotype supported by glioma, thus promoting the progression of glioma [10]. Besides, some myeloid-derived suppressor cells exist in glioma tissues. In the *in vitro* research model of glioma, studies have found that dendritic cells, fibroblasts, and macrophages in the tumour microenvironment can accelerate tumour growth through malignant transformation [11].

Immunogenic cell death (ICD), as an important mechanism of immunotherapy, can be induced by a variety of chemicals, radiation, and targeted drugs, releasing a variety of damage-associated molecular patterns (DAMPs), which can destroy the immunosuppressive tumour microenvironment and re-establish the body’s tumour cell immune monitoring, so as to play an anti-tumour role through systemic anti-tumour immunity [12]. Hossain et al. found that after inducing ICD to kill tumour cells, inoculating dead tumour cells into mice can establish effective immune response and prevent subsequent tumour growth [13]. Another experiment showed that low-dose chemotherapy combined with radiotherapy could better induce ICD in tumour cells, produce significant anti-tumour efficacy and prolong survival time [14]. GBM has a high degree of malignancy, and radiotherapy and chemotherapy do not significantly improve the prognosis of patients [15,16]. Moreover, the effect of immunotherapy on GBM remains controversial. Therefore, new personalised treatment strategies are required to improve its biological behaviour. This study aims to construct a personalised ICD-related score to assess GBM prognosis and the effect of immunotherapy.

## 2 Materials and methods

### 2.1 Defining ICD-related regulators and data processing

ICD-related genes (Table S1) were identified and extracted from a previous study, which included 33 ICD-related genes [17]. Data were extracted from The Cancer Genome Atlas (TCGA), Chinese Glioma Genome Atlas (CGGA) [18], and Gene Expression Omnibus (GEO) databases. Datasets GSE74187 [19] and GSE83300 [20] from the GEO database contained information related to the prognosis of patients with GBM. RNA sequencing data (RNA-seq) were downloaded for normalising and log2 conversion along with the related clinical information of patients with GBM. Samples with missing clinical data and no clear diagnosis of GBM were excluded. Based on a previous study, the expression profiles (Fragments Per Kilobase of exon model per Million mapped fragments values) of the TCGA-GBM dataset were converted to millions of transcripts per kilobase, which provides better results than microarray analysis and aids in better merging [21]. Furthermore, the “ComBat” algorithm was applied to reduce the possibility of batch effect caused by the non-biotechnology deviation between the different datasets [22].

### 2.2 Unsupervised clustering and identification of differentially expressed genes (DEGs)

After merging the datasets, unsupervised consensus clustering analysis was performed to obtain different ICD clusters based on ICD-associated RNA-seq for each GBM sample. “ConsensuClusterPlus” R package was used, and the analysis was repeated 1,000 times to ensure classification stability. Based on the difference between different ICD clusters, the cut-off criterion of significance was set at adjusted *p* < 0.05 and absolute fold change >0.75 to obtain DEGs using the “limma” R package. The DEGs underwent unsupervised consensus clustering analysis to obtain different gene clusters.

### 2.3 Construction of tumour-infiltrating immune cells

Twenty-two types of tumour immune infiltration-related lymphocytes were identified using the Cell-type Identification By Estimating Relative Subsets Of RNA Transcripts algorithm [23,24]. Additionally, the Estimation of Stromal and Immune cells in MAlignant Tumours using Expression data algorithm was used to evaluate the immune and stromal scores for each GBM sample [25].

### 2.4 Construction of ICD scores

Patients with GBM were classified based on DEG values using the unsupervised clustering method. Additionally, the DEG values that were positively and negatively correlated with the clustering signature were named ICD gene signatures A and B, respectively. Dimensionality reduction was subsequently performed on the ICD gene signatures A and B using the Boruta algorithm [26]. Principal component 1 was extracted as signature scores using principal component analysis (PCA) [27,28]. Finally, we applied a method similar to the Gene Expression Grading Index to define the ICD score for each patient with GBM: ICD score = ∑PC1_B_ – ∑PC1_A._

### 2.5 Collection of somatic alteration data

The corresponding mutation data for patients in the TCGA-GBM cohort were obtained from the TCGA database. To determine the mutational burden of GBM, we counted the total number of non-synonymous mutations in GBM samples and assessed somatic changes in GBM driver genes based on high or low ICD scores. HNSC driver genes were identified using the “maftool” R package [29]. The top 27 driver genes with the highest mutation frequency were further analysed.

### 2.6 Immunotherapy-related gene expression data

Two datasets (IMvigor210 [30] and GSE78220 [31]) containing immunotherapy effect and prognosis-related information were used for external verification. The IMvigor210 cohort is a single-arm phase II clinical study of the PD-L1 monoclonal antibody atezolizumab in locally advanced or metastatic urothelial carcinoma after the failure of platinum-based chemotherapy. GSE7820 is a dataset of 26 patients with PD-1-treated melanoma. The datasets contained RNA-seq data along with follow-up information and immunotherapy effects.

### 2.7 Statistical analysis

All statistical analyses were performed using the R software. The Kruskal–Wallis test was used to compare more than two groups, whereas the Wilcoxon test was used for comparison between two groups. Survival curves were generated for subgroups in each dataset using a Kaplan–Meier plotter, and statistically significant differences were assessed using the log rank test. The X-tile software was used to divide the best cut-off values into different subgroups [32]. Univariate and multivariate cox regression analyses were used to determine whether the ICD score was an independent prognostic factor. Gene ontology (GO) analysis and Gene Set Enrichment Analysis (GSEA) were used to determine the potential enriched biological functions or pathways. The independent prognostic factors obtained via multivariate cox regression analysis were used to construct a nomogram, and the time-dependent receiver operating characteristic (ROC) curve was used to assess the predictive ability of the nomogram. The Chi-square test analysed the correlation between ICD score subgroups and somatic mutation frequency, and Spearman analysis calculated the correlation coefficient. *p* < 0.05 was statistically significant.

## 3 Results

### 3.1 Data processing

**Figure 1:**
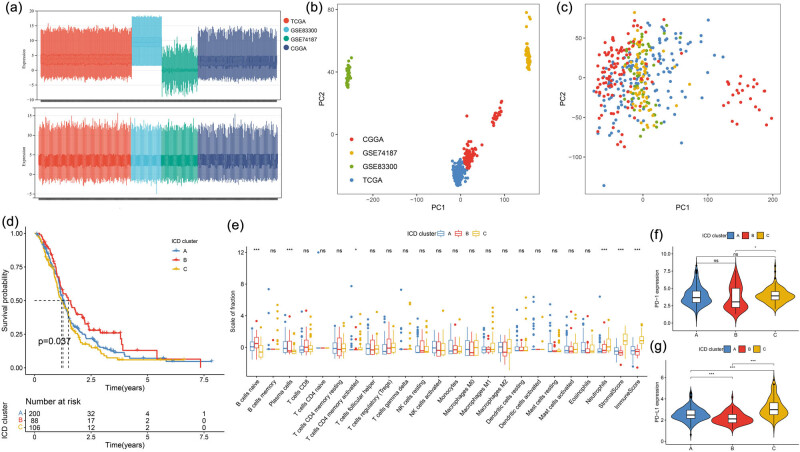
(a) Box plot showing the overall profiles of the TCGA, GSE83300, GSE74187, and CGGA datasets before and after normalisation. PCA of the overall profiles (b) before normalisation and (c) after normalisation. (d) Kaplan–Meier curve of the different ICD clusters, log rank test showed an overall *p* = 0.037. (e) Box plot comparing 22 TILs and immune and stromal scores between different ICD clusters. Comparison of (f) PD-1 expression and (g) PD-L1 among patients in different ICD clusters, Kruskal–Wallis test. *p < 0.05; **p < 0.01; ***p < 0.001; ****p < 0.0001.

A total of 394 patients with GBM from four datasets were included in this study. As shown in Figure 1a, the boxplot reveals significant differences in the sample distributions of the four datasets before removing the batch effect, suggesting the existence of the batch effect. The distribution of data between the two datasets was observed to be consistent, and the median was on a horizontal line. Similarly, the PCA plot showed that the samples of each dataset were scattered before removing the batch effect (Figure 1b), suggesting the presence of an obvious batch effect. After removing the batch effect (Figure 1c), the samples between the various datasets were intertwined with each other, indicating the complete removal of batch effects.

### 3.2 Identification of ICD-related clusters

Three ICD clusters (Figure S1a) with significant differences in prognosis were obtained using unsupervised consistent clustering analysis. As shown in Figure 1d, the prognosis of patients in cluster B was the best, followed by cluster A and cluster C, respectively. No significant difference was observed in the tumour infiltrating lymphocytes (TILs) among the three clusters, except for naive B cells, plasma cells, activated CD4 memory T cells, and neutrophils. Furthermore, cluster A had the highest plasma and lowest neutrophil levels; cluster B had the highest naïve B cell levels; and cluster C had the highest level of activated CD4 memory T cells. Moreover, both immune and stromal scores were highest in cluster C and lowest in cluster B (Figure 1e). Furthermore, the expression of two immune checkpoints related to tumour immunotherapy was assessed in the three clusters. For PD-1, the patients in cluster C had higher expression levels than those in cluster B; however, no significant difference between clusters A and B and clusters A and C was observed (Figure 1f). For PD-L1, there were significant statistical differences among the three clusters. The patients in cluster C had the highest expression level, while those in cluster B had the lowest expression level (Figure 1g).

### 3.3 Identification of ICD-related gene clusters

**Figure 2:**
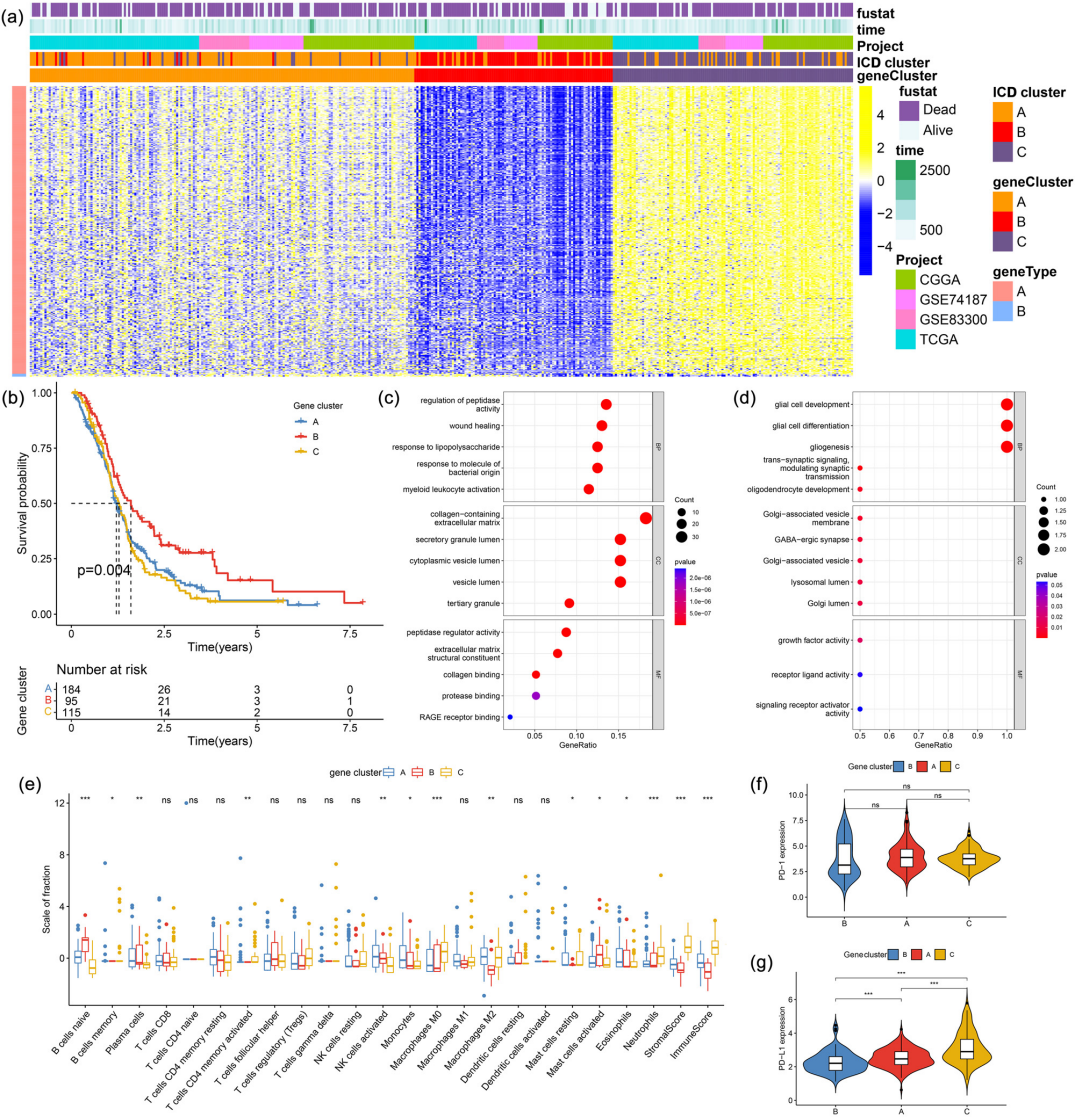
(a) Heatmap of DEGs. (b) Kaplan–Meier curve of different ICD gene clusters, log rank test showed an overall *p* = 0.004. Gene ontology analysis of (c) gene signature A and (d) gene signature B. (e) Box plot comparing 22 TILs and immune and stromal scores between different ICD gene clusters. Comparison of (f) PD-1 expression and (g) PD-L1 among patients in different ICD gene clusters, Kruskal–Wallis test. *p < 0.05; **p < 0.01; ***p < 0.001; ****p < 0.0001.

We obtained 307 DEGs (Table S2) and three ICD gene clusters (Figure S1b) with different prognoses. The heatmap of the distribution of these DEGs is shown in Figure 2a. Notably, ICD gene cluster B exhibited the best prognosis (Figure 2b). GO analysis revealed that gene signature A was the most enriched in myeloid leukocyte activation, collagen-containing extracellular matrix, and peptidase regulator activity (Figure 2c). Additionally, gene signature B was the most enriched in glial cell development, Golgi-associated vesicle membrane, and growth factor activity (Figure 2d). Gene cluster A had the largest proportion of memory B cells, activated natural killer cells, monocytes, resting mast cells, and eosinophils; gene cluster B had the largest proportion of naive B cells and activated mast cells; and gene cluster C had the largest proportion of activated CD4 memory T cells, M0 macrophages, and M2 macrophages. Notably, both immune and stromal scores were highest in gene cluster C and lowest in gene cluster B (Figure 2e). Moreover, there was no significant difference in the expression of PD-1 among the three gene clusters (Figure 2f). For PD-L1, patients in gene cluster C had the highest expression, followed by gene cluster A and then gene cluster B (Figure 2g).

### 3.4 Construction of ICD scores

**Figure 3:**
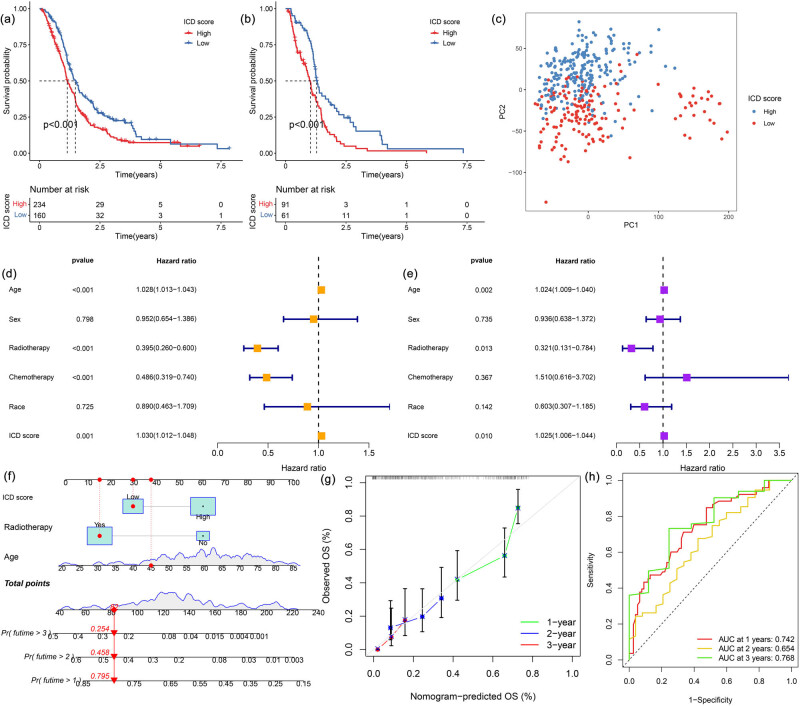
Kaplan–Meier curve of patients with GBM having different ICD scores in the (a) merged and (b) TCGA cohorts. (c) PCA of patients with GBM having low or high ICD scores. Results of (d) univariate and (e) multivariate Cox regression analyses. (f) Nomogram containing the independent prognostic factors score. (g) Calibration curve. (h) The results of the time-dependent ROC curve.

The dimensionality reduction of ICD-related gene signatures A and B was achieved using the Boruta algorithm, and PCA was used to extract the principal component 1 as the ICD score. The prognosis of patients with GBM with high ICD scores was significantly worse than those with low ICD scores (Figure 3a). In the TCGA cohort, patients with high ICD scores also had poor prognoses (Figure 3b). PCA analysis indicated that patients with GBM having high or low scores were well differentiated. As the TCGA cohort had more clinical information on patients and the largest sample size among the four datasets, we used the data from the TCGA database for subsequent analyses. Univariate and multivariate Cox regression analysis showed that ICD scores, age, and radiotherapy were independent prognostic factors, with a high ICD score indicating a worse prognosis (Figure 3d and e). The nomogram containing these factors is presented in Figure 3f. The calibration curve (Figure 3g) and time-dependent ROC curve (Figure 3h) also validate the good predictability of the nomogram.

### 3.5 Relationship of the ICD scores with somatic variants

**Figure 4:**
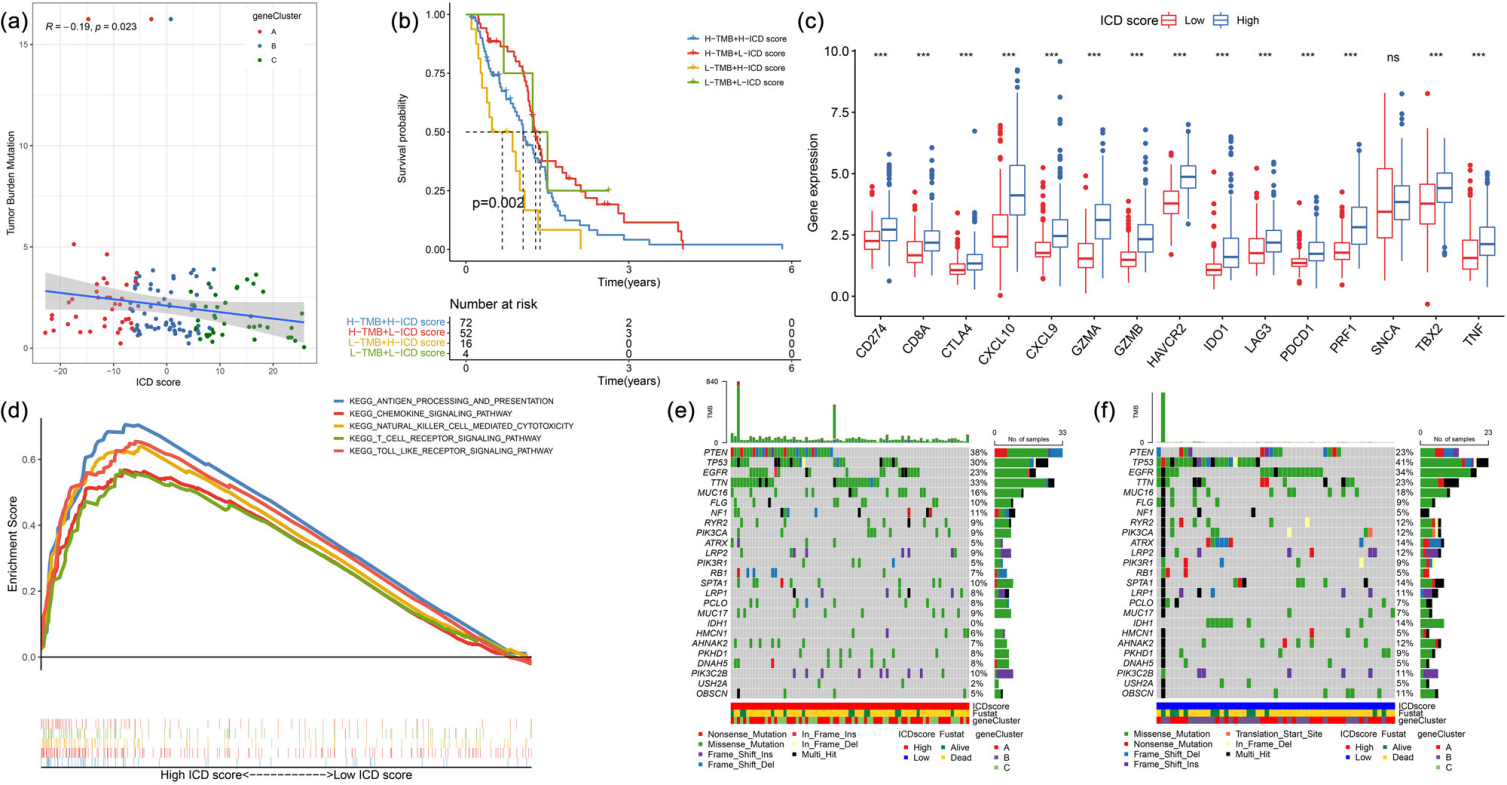
(a) The results of Spearman correlation analysis between ICD scores and TMB. (b) Kaplan–Meier curves of patients with GBM in the TCGA cohort stratified by TMB and ICD scores, log rank test showed an overall *p* = 0.002. (c) The expression level of immune-checkpoint-relevant and immune-activity-related genes in different ICD score groups. (d) The results of gene set enrichment analysis for high ICD score groups. A waterfall plot of (e) high ICD score and (f) low ICD score. *p < 0.05; **p < 0.01; ***p < 0.001; ****p < 0.0001.

A negative correlation was observed between the ICD score and Tumour Mutation Burden (TMB) (Figure 4a). The distribution of GBM patients in different ICD score and TMB is represented in Figure S2. TMB is a biomarker of immunosuppressive agents in patients with tumours and is related to prognosis. Therefore, TMB and ICD scores were considered prognostic factors for grouping analysis. Patients with GBM having low TMB and high ICD scores had the worst prognosis while those with high TMB scores and low ICD had the best prognosis (Figure 4b). Additionally, we analysed 15 immune-checkpoint-relevant and immune-activity-related genes [31,33], wherein patients with higher ICD scores had higher levels of expression of these genes except *SNCA* (Figure 4c). GSEA indicated that the group with a high ICD score was most enriched in antigen processing and presentation, natural killer cell-mediated cytotoxicity, and T cell receptor signalling pathways (Figure 4d). However, there was no significant enrichment of biological pathways in the low ICD score group. Using “maftools,” GBM driver genes were visualised, and the first 27 driver genes with the highest change frequency were further analysed (Figure 4e and f). The analysis of mutation annotation files of the TCGA database showed that the frequency of *IDH1* mutation was significantly different between the low and high ICD score groups (Table S3).

### 3.6 Roles of ICD scores on the effect of immunotherapy

**Figure 5:**
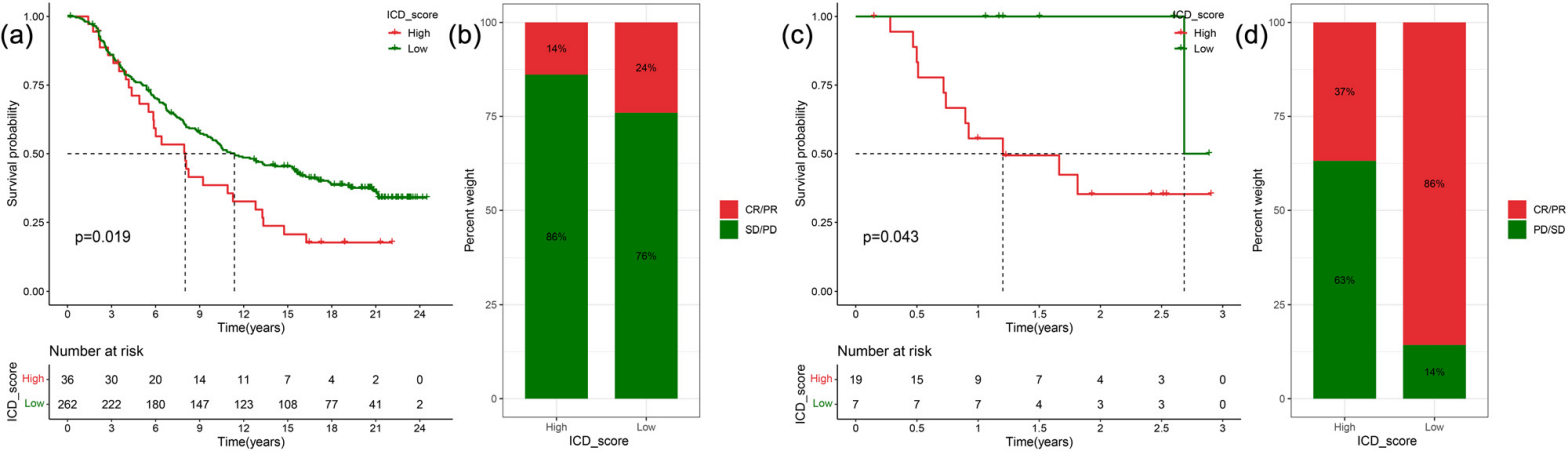
In the IMvigor210 cohort: (a) Kaplan–Meier curves of patients with high and low ICD scores. (b) Rate of clinical response to immunotherapy. In the GSE78220 cohort: (c) Kaplan–Meier curves of patients with high and low ICD scores. (d) Rate of clinical response to immunotherapy.

Two independent datasets were used for the external validation of ICD scores in relation to prognosis and immunotherapy effect. In the IMvigor210 cohort, the prognosis of patients with a high ICD score was significantly worse than those with a low ICD score (Figure 5a). The proportion of patients with complete response (CR) and partial response (PR) to immunotherapy in the low ICD group was significantly higher than that in the high ICD group (Figure 5b). In the GSE78220 cohort, the prognosis of patients with a high ICD score was significantly worse than that of those with a low ICD score (Figure 5a). Furthermore, the proportion of patients with CR and PR to immunotherapy in the low ICD group was significantly higher than that in the high ICD group (Figure 5b).

## 4 Discussion

ICD stimulates the immune system to generate immune responses by releasing tumour-related antigens and tumour-specific antigens [34,35]. It is characterised by the increase in the expression of DAMPs, precursor antigens, inflammatory cytokines, and inflammatory mediators [35]. As a highly malignant tumour of the nervous system, GBM responds poorly to radiotherapy and chemotherapy and has a poor prognosis. Nonetheless, immunotherapy is speculated to have a relatively better effect on GBM than other conventional treatments [36]. Therefore, personalised precise treatments are essential for patients with GBM. Recently, a few studies have identified biomarkers of glioma using bioinformatics analysis [37,38] and developed gene-related signatures based on sequencing data to assess the prognosis of patients with tumours and the effect of immunotherapy [28,39,40]. Considering the correlation between ICD and tumour immunotherapy, herein we constructed an ICD-related signature to assess the prognosis of patients with GBM and their response to immunotherapy. Additionally, validation using two independent external datasets revealed that the signature has a good predictive ability.

With the rapid development of bioinformatics technology and the popularity of sequencing technology, there is an increasing number of research works on the use of sequencing data to construct prognosis or diagnosis-related signatures. Song et al. used the data from TCGA and to construct a necroptosis-related lncRNA-related signature that can be used to judge the prognosis of IDH wild-type GBM patients and the ACU of the signature is between 0.65 and 0.7 [41]. Zhao et al. also constructed a four-gene signature and the AUC of the signature was 0.587–0.701 when the patients using the TCGA database were used for prognosis analysis, and was 0.655–0.955 when the CGGA database was used for validation [42]. Yuan et al. constructed a gene signature related to subventricular zone, whose AUC in the CGGA database was 0.635–0.739, and in the TCGA database was 0.678–0.737 [43]. Since the sample sizes in GBM-related databases are relatively small compared to other tumours, the signatures constructed by most studies are not very good for prognostic judgment. In this study, we used the sequencing data of GBM patients from three different databases to construct an ICD-related signature, which has an AUC of more than 0.7 in predicting prognosis and can also be used to reflect patients’ response to immunotherapy.

TILs are considered to be one of the factors affecting the prognosis of patients with tumours. In this study, immune cells in the different ICD clusters and gene clusters exhibited statistical differences. Additionally, GSEA also revealed that gene sets with high ICD scores were enriched in specific pathways related to immune cells. Owing to the immunosuppressive microenvironment, lack of tumour-specific antigen expression, and other factors, GBM remains insensitive to immunotherapy and is considered a “cold tumour.” Compared to other types of tumours, GBM has fewer TILs, which indicates the presence of inertia in the immune response [44]. However, the association between TILs in patients with GBM and prognosis remains unclear [2,45,46,47]. Nonetheless, there exist various immunosuppressive factors in GBM that should be explored further to improve the immunosuppressive microenvironment of GBM and enhance the efficacy of immunotherapy.

ICD score was observed to be an independent prognostic risk factor for patients with GBM in this study, indicating that ICD could be related to the promotion of tumour cell inactivation. A previous study reported that ferroptosis-related scores were related to the prognosis of patients with lung squamous cell carcinoma [28]. Similarly, in the TCGA cohort, we observed that patients with GBM having *IDH1* mutations were higher in number than those with high ICD scores. Moreover, *IDH1* showed a significant difference between the two ICD groups. Studies have shown that *IDH1* mutation plays an important role in the diagnosis and prognosis of diffuse glioma [48,49,50]. Accordingly, *IDH1* status, a defining feature of different tumours, has been included in the WHO Central Nervous System Tumour Classification in 2016 [51]. In the retrospective analysis of 382 cases of anaplastic astrocytoma and glioblastoma by Hartmann et al., it was proved that *IDH1* mutation was a stronger predictor of OS than histological grading [52]. Furthermore, IDH-mutated tumours also showed a good response to chemotherapy. Randomised clinical trials in patients with glioma [53,54] receiving radiotherapy showed that only patients having *IDH1* mutated tumours benefited significantly from radiotherapy combined with adjuvant chemotherapy. Thus, this supports the hypothesis that ICD scores can be used to assess the prognosis of patients with GBM.

Currently, studies on the immunotherapy of GBM report that immune checkpoint PD-1 and PD-L1 antibodies have been used in the treatment of tumours with remarkable effects. However, the effect of tumour immunotherapy is not beneficial to all patients and requires personalisation for each tumour. A study reports that the PD-1 antibody nivolumab can delay the progress of GBM recurrence [55]. Additionally, studies also report that patients with high PD-L1 expression in GBM have a worse prognosis, which could be related to its immunosuppressive mechanisms [56]. A recent meta-analysis of 2,943 patients with GBM in 19 studies also suggests that the high expression of PD-L1 indicates worse OS [57]. Consistent with previous studies, we also observed that PDL1 (CD274) was highly expressed in patients with high ICD scores in the TCGA cohort. Patients with high ICD score have poor immunotherapy effect, which may be because the score we constructed is determined by the expression of genes, and some ICD-related genes may inhibit ICD. Also, a signature of four ICD-related genes was constructed and found that patients with high ICD scores were less likely to respond to immunotherapy [58].

TMB refers to the total number of somatic non-synonymous mutations with an average of 1 Mb base in the exon region after excluding the germline mutations from the genome [59]. The higher the tumour TMB, the higher the level of antigens produced. Moreover, the higher the tumour immunogenicity, the higher the T cell anti-tumour response [60]. Research shows that TMB level is strongly correlated with the objective response rate of PD-1/PD-L1 inhibitor treatment in multiple tumour species. The level of TMB was also positively correlated with the OS of patients undergoing immunotherapy [61,62]. However, the association between TMB and the effect of GBM immunotherapy requires further exploration. This study observed that the ICD score was weakly negatively related to TMB, and patients with a high TMB had a poor prognosis. Additionally, a higher percentage of patients with low ICD scores who responded effectively to immunotherapy was observed. Thus, the ICD score has the potential as an independent biomarker for predicting the immunotherapy effect.

Our research has some limitations. First, this study can be considered a secondary analysis as the sequencing data and clinical follow-up information used in this study were obtained from public databases. Although the prediction ability of ICD score for prognosis and immunotherapy effect has been verified using external data, it still lacks validation using sequencing data and relevant follow-up information. Second, the absolute value of the ICD score was generated using a dimensionality reduction process of gene expression data; therefore, our model emphasises the factor itself rather than the numerical value. Thus, a normalisation method should be developed for the clinical application of our study. Finally, the small sample size, with only a few hundred patient data, limits the generalisation of the study results. Furthermore, the validation dataset for predicting the effect of immunotherapy did not include GBM samples.

## 5 Conclusion

We constructed a signature related to the ICD and revealed that GBM patients with high ICD scores have a poor prognosis and may not respond to immunotherapy. Thus, this study provides a novel research direction for developing personalised treatment strategies for patients with GBM.

## Supplementary Material

Supplementary material
