# Supplementary material for: Genomic analysis of immunogenic cell death-related subtypes for predicting prognosis and immunotherapy outcomes in glioblastoma multiforme
Source: Open Med (Wars). 2023 Jun 2;18(1):20230716. doi: 10.1515/med-2023-0716 (PMC10238813; doi:10.1515/med-2023-0716)
Supplement: Supplementary material [file med-2023-0716-sm.pdf]

Supplementary material

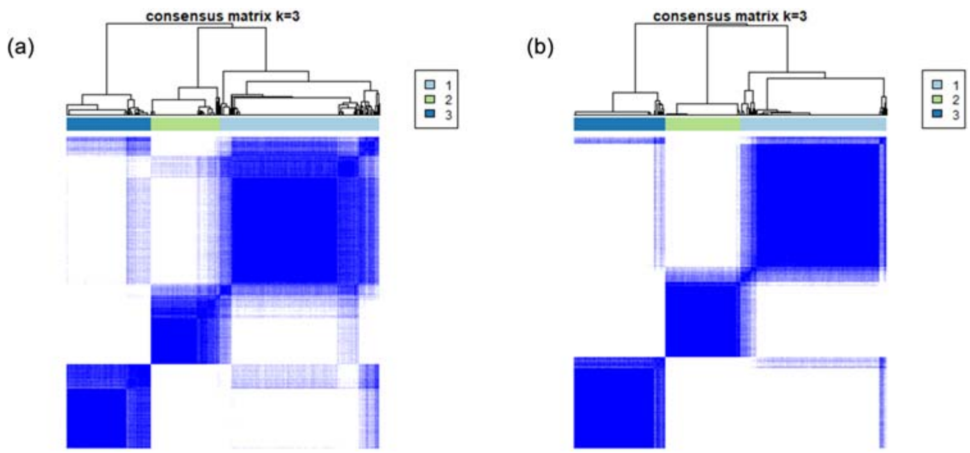

Figure S1: Consensus matrix heat map of (a) clusters and (b) gene cluster.

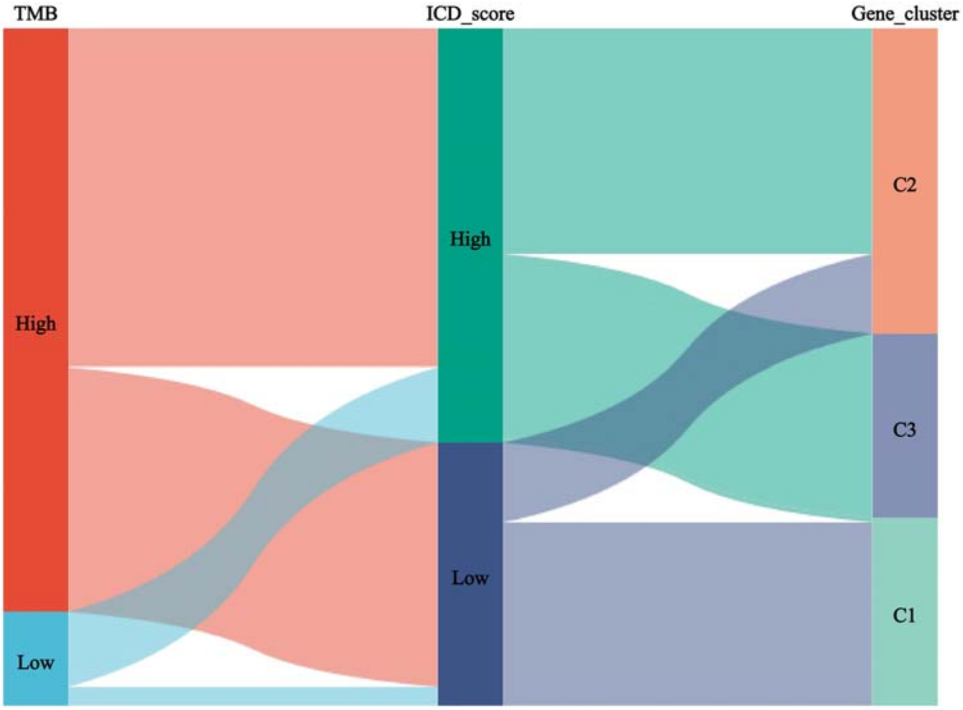

Figure S2: Alluvial diagram of ICD gene cluster distribution in groups with different TMB and ICD score.

Table S1: The ICD related genes

IL17RA
IL1R1
PIK3CA
CD4
IFNG
PRF1
CXCR3
CD8A
CD8B
P2RX7
NLRP3
IL10
TLR4
ENTPD1
ATG5
IFNB1
IL6
EIF2AK3
IL17A
LY96
FOXP3
HMGB1
HSP90AA1
BAX
PDIA3
CALR
CASP8
MYD88
IFNGR1
CASP1
IL1B
TNF
NT5E

Table S2: The DEGs of the ICD clusters

ARHGDIB	CASP1	S100A11	CASP4	CARD16	GPR65	NPC2	MNDA	LY96	VAMP8	FCER1G
SERPINB1	HLA-DRA	TLR2	GMFG	SAMSN1	S100A10	HLA-DMA	FCGR3A	SRGN	TNFSF13B	C1orf162
IL18	SERPINA1	SERPING1	VAMP5	ANXA2	CLIC1	ALOX5AP	LAPTM5	HLA-DPA1	CD53	CPVL
S100A4	GLRX	CCR1	AIF1	HEXB	CD74	CSTA	CFI	C1S	HLA-B	C3AR1
SPP1	ANXA1	IFITM3	ALOX5	ARPC1B	ISG20	PTN	HLA-DMB	TYROBP	FCGR1B	CLEC2B
SLC7A7	CCR5	CREG1	CD33	FERMT3	MS4A6A	TIMP1	FXYD5	GBP2	CTSS	CXCR4
C1R	PLEK	SAT1	IFITM2	BATF	HLA-DRB1	PTPRC	CTSC	SPI1	PYCARD	RAC2
DEF6	FUCA1	MYO1F	CYBA	CEBPD	HLA-DPB1	FCGR2A	PLBD1	LILRB1	RNASET2	CYTIP
CLEC7A	HCLS1	IL32	NCF4	C1QB	TRPV2	LCP2	C3	RELB	CD48	DENND2D
CAPG	CFH	PLAU	HLA-F	MS4A4A	NCF1	TGFBI	LAIR1	TNFAIP8L2	CHI3L2	HCST
TNFRSF14	PTPN6	HCK	FBP1	MAN1C1	CD44	GLIPR1	LST1	C1QC	MILR1	VAV1
RRAS	CTSB	CCL2	MS4A7	STEAP3	LY86	WAS	HAVCR2	PDPN	GPSM3	COPZ2
MSR1	CD37	RNASE6	CP	GPR183	IFI30	TNFSF10	SLC15A3	FBLN5	SERPINA3	ZFP36
NAMPT	LCP1	UPP1	MVP	TYMP	RNASE2	LILRB4	GPX8	ITGB2	EFEMP1	LGALS3
DAB2	LILRB2	MAP3K8	CD68	LTBR	RUNX1	CYBB	BST2	TMEM176A	CEBPB	LUM
FTL	IGFBP4	NUPR1	SCIN	ARHGAP4	CXCL10	HLA-DOA	SYNGR2	GZMA	SECTM1	CD248
POLD4	MGP	DUSP23	LGMN	PLTP	C1RL	CLEC5A	CCL5	TMEM176B	FAM20C	APOL1
FN1	TCIRG1	LAMB1	CD86	FAM20A	PLP2	CHI3L1	VASN	MYL9	CTSD	MT2A
CSF1R	UBD	PDLIM1	SLAMF8	HAMP	FABP5	IFITM1	NNMT	PRSS23	SRPX2	PLAUR
SOD2	BCL2A1	HMOX1	FPR1	CAV1	ASPN	BCL3	CD14	VSIG4	FTH1	IER3
IL4I1	CD52	GPNMB	BIRC3	C1QA	HLA-DQB1	GBP5	PTX3	COL18A1	TUBA1C	CD69
PLIN2	SLC11A1	FAP	TNFAIP2	LYZ	LOXL1	DCN	S100A8	ACTA2	CD93	MT1G
RDH10	IBSP	PCOLCE	TAGLN	KCNE4	MT1L	SLC47A2	RCN3	BHLHE40	MGST1	SOD3
CD3D	FCGBP	HSPA6	SERPINF1	RGS1	CTSH	IGFBP3	GADD45B	EMILIN1	TDO2	CH25H
ABCC3	HTRA3	SPON2	HLA-DRB5	CFD	CXCL2	CD163	FMOD	COL6A2	GDF15	LTF
CTHRC1	POSTN	SAA1	S100A9	LOX	FAM183A	SLPI	SPOCD1	MMP9	ACTG2	G0S2
RARRES2	ESM1	MYBPH	HLA-DQA1	GJB2	CA12	COL1A2	PDLIM4	COL1A1	CSPG5	MOXD1
SULF1	CXCL14	ANGPTL4	CCL3	IL13RA2	OLIG1	SAA2	PLA2G2A	COL3A1	SLN	0

Table S3: Comparison of ICD scores with somatic variants

Gene	H-wild	H-mutation	L-wild	L-mutation	*p* value
IDH1	88(100%)	0(0%)	48(85.71%)	8(14.29%)	0.00105557
ATRX	84(95.45%)	4(4.55%)	48(85.71%)	8(14.29%)	0.079708361
PTEN	55(62.5%)	33(37.5%)	43(76.79%)	13(23.21%)	0.107604209
EGFR	68(77.27%)	20(22.73%)	37(66.07%)	19(33.93%)	0.199767932
TP53	62(70.45%)	26(29.55%)	33(58.93%)	23(41.07%)	0.213976893
OBSCN	84(95.45%)	4(4.55%)	50(89.29%)	6(10.71%)	0.27863905
TTN	59(67.05%)	29(32.95%)	43(76.79%)	13(23.21%)	0.286618795
NF1	78(88.64%)	10(11.36%)	53(94.64%)	3(5.36%)	0.353475609
AHNAK2	82(93.18%)	6(6.82%)	49(87.5%)	7(12.5%)	0.388911802
PIK3R1	84(95.45%)	4(4.55%)	51(91.07%)	5(8.93%)	0.480071317
USH2A	86(97.73%)	2(2.27%)	53(94.64%)	3(5.36%)	0.603947276
SPTA1	79(89.77%)	9(10.23%)	48(85.71%)	8(14.29%)	0.637711486
COL6A3	83(94.32%)	5(5.68%)	51(91.07%)	5(8.93%)	0.68111809
RYR2	80(90.91%)	8(9.09%)	49(87.5%)	7(12.5%)	0.709105093
PIK3CA	80(90.91%)	8(9.09%)	49(87.5%)	7(12.5%)	0.709105093
LRP2	80(90.91%)	8(9.09%)	49(87.5%)	7(12.5%)	0.709105093
LRP1	81(92.05%)	7(7.95%)	50(89.29%)	6(10.71%)	0.790928281
DNAH5	81(92.05%)	7(7.95%)	53(94.64%)	3(5.36%)	0.793702385
MUC17	80(90.91%)	8(9.09%)	52(92.86%)	4(7.14%)	0.91789851
MUC16	74(84.09%)	14(15.91%)	46(82.14%)	10(17.86%)	0.939063416
SYNE1	84(95.45%)	4(4.55%)	53(94.64%)	3(5.36%)	1
DNAH2	84(95.45%)	4(4.55%)	53(94.64%)	3(5.36%)	1
PCLO	81(92.05%)	7(7.95%)	52(92.86%)	4(7.14%)	1
PKHD1	81(92.05%)	7(7.95%)	51(91.07%)	5(8.93%)	1
PIK3C2B	79(89.77%)	9(10.23%)	50(89.29%)	6(10.71%)	1
FLG	79(89.77%)	9(10.23%)	51(91.07%)	5(8.93%)	1
HMCN1	83(94.32%)	5(5.68%)	53(94.64%)	3(5.36%)	1
